# A New Butanolide Compound from the Aerial Part of *Lindera akoensis* with Anti-inflammatory Activity

**DOI:** 10.3390/molecules17066585

**Published:** 2012-05-31

**Authors:** Chung-Ping Yang, Guan-Jhong Huang, Hui-Chi Huang, Yu-Chang Chen, Chi-I Chang, Sheng-Yang Wang, Ih-Sheng Chen, Yen-Hsueh Tseng, Shih-Chang Chien, Yueh-Hsiung Kuo

**Affiliations:** 1 School of Chinese Pharmaceutical Sciences and Chinese Medicine Resources, China Medical University, Taichung 404, Taiwan; 2 Department of Biological Science and Technology, National Pingtung University of Science and Technology, Pingtung 912, Taiwan; 3 Department of Forestry, National Chung Hsing University, Taichung 402, Taiwan; 4 Agricultural Biotechnology Research Center, Academia Sinica, Taipei 115, Taiwan; 5 Graduate Institute of Natural Products, Kaohsiung Medical University, Kaohsiung 807, Taiwan; 6 The Experimental Forest Management Office, National Chung-Hsing University, Taichung 402, Taiwan; 7 Tsuzuki Institute for Traditional Medicine, China Medical University, Taichung 404, Taiwan

**Keywords:** Chinese medicinal herbs, *Lindera akoensis*, butanolide, anti-inflammatory

## Abstract

A new butanolide, 3β-((*E*)-dodec-1-enyl)-4β-hydroxy-5β-methyldihydrofuran-2-one (**1**) and four known butanolides: Akolactone A (**2**), (3*Z*,4α,5β)-3-(dodec-11-enylidene)-4-hydroxy-5-methylbutalactone (**3**), (3*E*,4α,5β)-3-(dodec-11-enylidene)-4-hydroxy-5-methylbutalactone (**4**) and dihydroisoobtusilactone (**5**), were isolated from the aerial parts of *Lindera akoensis.* These butanolides showed *in vitro* anti-inflammatory activity decrease the LPS-stimulated production of nitrite in RAW264.7 cell, with IC_50_ values of 1.4–179.9 μM.

## 1. Introduction

*Lindera akoensis* (Lauraceae) is an endemic evergreen tree that grows in broad-leaved forests in lowlands throughout Taiwan, it is often used as a hedge. Aporphines [1], alkaloids [2], sesquiterpenoids [3,4,5], flavanoids [6], butanolides [6], furanoids [7], chalconoids [8], and phenolic compounds [9,10] are widely distributed in the plants of the genus *Lindera.* Some isolates exhibit biological activities, including suppressed contraction of thoracic aorta [1], anti-mycobacterial [6], anti-inflammatory [11], anti-human lung cancer cell (SBC-3) [12], osteoclast differentiation inhibitory [10], slowing done of the progression of diabetic nephropathy in mice [12], anti-nociceptive [13], human acyl-CoA:cholesterol acyltransferase inhibitory activity, and low density lipoprotein anti-oxidation effects [9]. Only one reference has reported the chemical constituents and anti-mycobacterial activity until now from the root of *L. akoensis* [6].

The folk usage of *L. akoensis* is in the treatment of trauma and inflammation [14]. Butanolides showed anti-inflammation in previous studies [15,16]. In a random screening for inhibitory activity of various Chinese traditional medicines toward nitric oxide (NO) production *in vitro* by RAW264.7 cells, the EtOH extract of the aerial parts of *L. akoensis* showed a significant activity. Thus, the constituents of *L. akoensis* were investigated. This paper deals with the structure elucidation of the new compound and the inhibitory activity of the isolates toward nitric oxide (NO) production and *in vitro* cytotoxicity towards RAW264.7 cells is also discussed.

## 2. Results and Discussion

**Figure 1:**
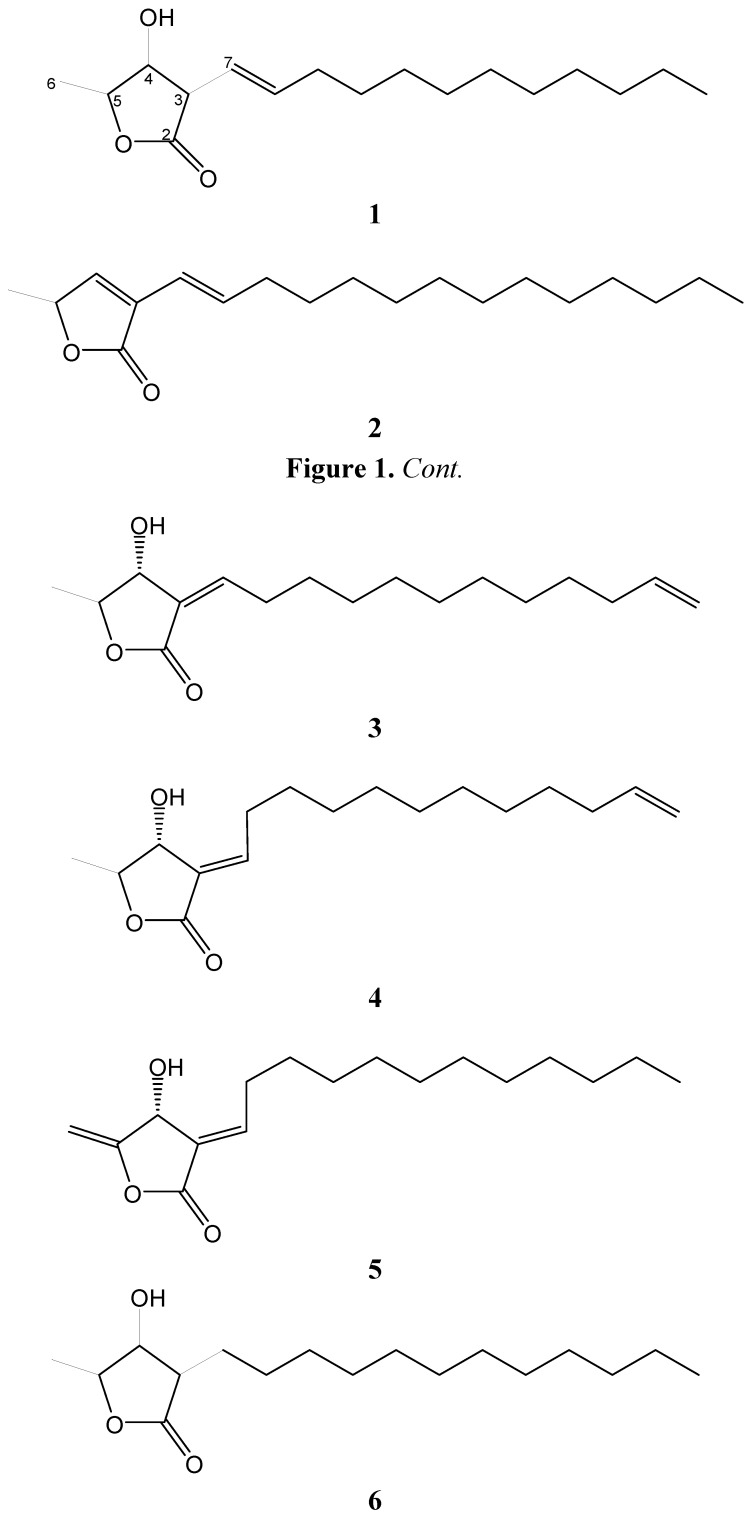
Structures of **1**–**6**.

The aerial parts of *L. akoensis* were air-dried and then extracted with EtOH and purified. Extensive normal phase Si gel column chromatographic purification of the EtOAc-soluble fraction afforded a new compound: 3β-((*E*)-dodec-1-enyl)-4β-hydroxy-5β-methyldihydrofuran-2-one (**1**), as well as four known compounds: akolactone A (**2**) [17], (3*Z*,4α,5β)-3-(dodec-11-enylidene)-4-hydroxy-5-methylbutalactone (**3**) [18], (3*E*,4α,5β)-3-(dodec-11-enylidene)-4-hydroxy-5-methylbutalactone (**4**) [17], dihydroisoobtusilactone (**5**) [17] (Figure 1).

**Figure 2:**
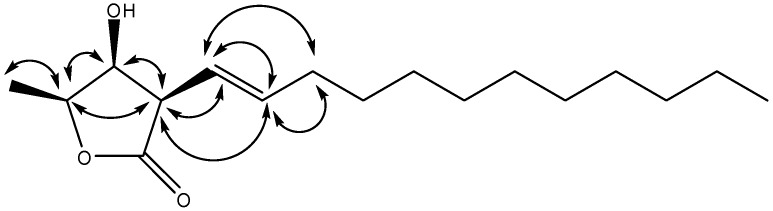
Significant NOESY correlations (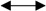) of **1**.

Compound **1** was isolated as a near to optically inactive pale yellow solid {[α]^20^_D_ ±0° (*c* 0.2, CHCl_3_)} that showed the presence of hydroxy (3,433 cm^−1^), olefin (1,681cm^−1^), and lactone (1,751 cm^−1^) functionalities in its IR spectrum. The HREIMS data determined the molecular formula to be C_17_H_30_O_3_ (*m/z* 282.2191 ([M]^+^; calcd. 282.2195)). The ^1^H-NMR spectrum showed signals at δ 3.37 (1H, *dd*, *J* = 6.5, 5.0 Hz), δ 4.27 (*dd*, *J* = 5.0, 3.2), δ 4.47 (*qd*, *J* = 6.5, 3.2) were assigned to H-3, H-4, and H-5, respectively (Table 1), those are similar to those of [(3β,4β,5β)-3-dodecyl-4-hydroxy-5-methyldihydrofuran-2-one] (**6**) [19]. The chemical shifts and coupling patterns of H-4 and H-5 suggested that the relative configuration of **1** was also identical to that compound **6**. This conclusion was supported by comparison of the ^1^H- and ^13^C-NMR data of **1** with those of reported compounds having a *cis*-relationship between H-4 and H-5. Two olefinic H-atoms were assigned the signals at δ 5.48 (1H, *dd*, *J* = 15.8, 6.5 Hz, H-7), δ 5.83 (1Η, *dt*, *J* = 15.8, 6.9 Hz, H-8), and nine CH_2_-group signals were observed [δ 2.11 (2H, *q*, *J* = 6.9 Hz, H-9) and δ 1.24 (16 Η, m, H-10~17)]. One set of contiguous H-atoms δ 3.37 (1H, *dd*, *J* = 6.5, 5.0 Hz), δ 4.27 (1H, *dd*, *J* = 5.0, 3.2), δ 4.47 (1H, *qd*, *J* = 6.5, 3.2) were assigned to H-3, H-4, and H-5, respectively, and these signals were also revealed by the COSY spectrum. The H-7 was coupled with H-3 and H-8, with coupling constant 6.5 Hz and 15.8 Hz, respectively, established the *trans*-geometry between H-7 and H-8. The key correlations in the NOESY spectrum (H-3 has correlation with H-4, H-5, and no correlation with H-6; H-4 have no correlation with H-6, H-7, Figure 2) confirmed the configuration of H-3, H-4 and H-5 in the same phase. All protons and carbons were confirmed by 1D and 2D spectra. Thus, **1** was identified as 3β-((*E*)-dodec-1-enyl)-4β-hydroxy-5β-methyldihydrofuran-2-one.

**Table 1:** NMR data (CDCl_3_) of **1**. δ in ppm, *J* in Hz.

No.	δ_H_^a^	δ_C_^b^
2	-	175.7
3	3.37 ( *dd*, *J* = 6.5, 5.0)	50.7
4	4.27 ( *dd*, *J* = 5.0, 3.2)	72.2
5	4.47 ( *qd*, *J* = 6.5, 3.2)	78.6
6	1.43 ( *d*, *J* = 6.5)	13.7
7	5.48 ( *dd*, *J* = 15.8, 6.5)	118.9
8	5.83 ( *dt*, *J* = 15.8, 6.9)	139.7
9	2.11 ( *q*, *J* = 6.9)	32.9
10–15	1.24 (m)	29.0–31.9
16–17	1.24 (m)	22.7
18	0.86 (t, *J* = 6.6)	14.1

^a^ Recorded at 400 MHz; ^b^ Recorded at 100 MHz.

Akolactone A (**2**) and (3*Z*,4α,5β)-3-(dodec-11-enylidene)-4-hydroxy-5-methylbutalactone (**3**), were isolated as light yellow oils, whereas (3*E*,4α,5β)-3-(dodec-11-enyl)-4-hydroxy-5-methylbutalactone (**4**) and dihydroisoobtusilactone (**5**) were obtained as light yellow solids. The ^1^H and ^13^C-NMR spectra of compounds **2**–**5** were compared with the spectral data reported in the literature confirming their structures. 

NO, produced from L-arginine by NO synthase, has various biological actions, e.g., as a defense and regulatory molecule for homeostatic equilibrium [20]. However, in pathophysiologic conditions, such as inflammation, there is an increased production of NO by inducible NO synthase (iNOS) [21]. Macrophages have been expected to be an origin of inflammation, because they contain various chemical mediators that may be responsible for several inflammatory stages [22]. The inhibitory activity toward NO production, induced by lipopolysaccharides (LPS), by murine macrophage-derived RAW264.7 cells was assayed. These compounds from *L. akoensis* were screened by anti-inflammatory activity *in vitro* with decrease nitrite of LPS-stimulated production in RAW264.7 cell with IC_50_ values of 1.4–179.9 μM and cell viability also be evaluated (Table 2).

**Table 2:** Cell viability and *in vitro* anti-inflammatory activities of compound **1**–**5** decrease nitrite of LPS-stimulated production in RAW 264.7 cell.

Compound	LD_50_ [μM]	IC_50_ [μM]
**1**	72.3	44.3
**2**	>211.9	179.9
**3**	49.3	2.4
**4**	67.1	1.4
**5**	>178.6	97.9
**Indomethacin**		182.9

## 3. Experimental

### 3.1. General

UV spectra were obtained with a Shimadzu Pharmaspec-1700 (Taichung, Taiwan) UV-Visible spectrophotometer. Optical rotations were obtained with a Jasco P-1020 (Taichung, Taiwan) polarimeter. Infrared spectra were obtained with a Shimadzu IRprestige-21 Fourier transform infrared spectrophotometer. 1D- and 2D-NMR spectra were recorded with a Bruker DRX-400 FT-NMR (Taichung, Taiwan) spectrometer. Mass spectrometric (HREIMS) data were generated at the Mass Spectrometry Laboratory of the Chung Hsing University (Taichung, Taiwan). Column chromatography was performed using Merck Si gel (30–65 μM; Taichung, Taiwan), and TLC analysis was carried out using aluminum pre-coated Si plates and the spots were visualized using a UV lamp at λ = 254 nm.

### 3.2. Collection, Extraction and Isolation

*Lindera akoensis* was collected and identified by Dr. Yen-Hsueh Tseng (Department of Forestry, National Chung Hsing University) at Taichung, Taiwan in July, 2008. The materials were totally dried in air under dark. The dried aerial parts of *L. akoensis* (5.9 kg) were cut into small pieces and soaked in 95% ethanol (60 L, 7 days × 3). After filtration, the crude extract was concentrated and stored under vacuum to yield an brown thick paste (337.8 g) that was suspended in H_2_O (1,000 mL) and extracted with ethyl acetate (1,000 mL, 3 times). The resulting ethyl acetate extract was concentrated to yield 127.8 g of an brown thick oil that was purified by 1,900 g silica gel with particle size 0.063–0.200 mm and internal diameter of column 15 cm packed height 25 cm chromatography with using a gradient of increasing polarity with *n*-hexane/ethyl acetate (99:1–1:99) as mobile phase and separated into 21 fractions on the basis of TLC analysis for random isolation of compounds. Fraction 8 (10.84 g) was re-separated by chromatography and semi-preparative HPLC with 20% EtOAc in *n*-hexane to afford pure butanolide **5** (20.4 mg), fraction 11 (5.08 g) was re-separated by chromatography and semi-preparative HPLC with 40% EtOAc in *n*-hexane to afford pure butanolide **1** (4.2 mg), **2** (4.4 mg), **3** (5.3 mg) and **4** (14.9 mg).

*3β-((E)-dodec-1-enyl)-4β-hydroxy-5β-methyldihydrofuran-2-one* (**1**). Yellow solid; m.p.: 75–77 °C; [α]^20^_D_ ±0° (*c* = 0.2, CHCl_3_); HREIMS *m/z:* 282.2191 [M]^+^ (calcd. for C_17_H_30_O_3_, 282.2195); IR (KBr)ν_max_: 3433, 1751, 1681, 1382 cm^−1^; ^1^H-NMR and ^13^C-NMR (400/100 MHz, in CDCl_3_): see Table 1. 

### 3.3. Chemicals

LPS (endotoxin from *Escherichia coli*, serotype 0127:B8), Carr (type IV), indomethacin, MTT (3-[4,5-dimethylthiazol-2-yl]-2,5-diphenyltetrazolium bromide) and other chemicals were purchased from Sigma Chemical Co. (St. Louis, MO, USA).

### 3.4. Cell Culture

A murine macrophage cell line RAW264.7 (BCRC No. 60001) was purchased from the Bioresources Collection and Research Center (BCRC, Hsinchu, Taiwan) of the Food Industry Research and Development Institute (Hsinchu, Taiwan). Cells were cultured in plastic dishes containing Dulbecco’s Modified Eagle Medium (DMEM, Sigma, St. Louis, MO, USA) supplemented with 10% fetal bovine serum (FBS, Sigma) in a CO_2_ incubator (5% CO_2_ in air) at 37 °C and subcultured every 3 days at a dilution of 1:5 using 0.05% trypsin-0.02% EDTA in Ca^2+^-, Mg^2+^-free phosphate-buffered saline (DPBS). 

### 3.5. Cell Viability

Cells (2 × 10^5^) were cultured in 96-well plate containing DMEM supplemented with 10% FBS for 1 day to become nearly confluent. Then cells were cultured with compounds **1**–**5** in the presence of 100 ng/mL LPS (lipopolysaccharide) for 24 h. After that, the cells were washed twice with DPBS and incubated with 100 μL of 0.5 mg/mL MTT for 2 h at 37 °C testing for cell viability. The medium was then discarded and 100 μL dimethyl sulfoxide (DMSO) was added. After 30-min incubation, absorbance at 570 nm was read using a microplate reader (Molecular Devices, Sunnyvale, CA, USA).

### 3.6. Measurement of Nitric Oxide/Nitrite

NO production was indirectly assessed by measuring the nitrite levels in the cultured media and serum determined by a colorimetric method based on the Griess reaction. The cells were incubated with butanolides (0, 3.125, 6.25, 12.5, 25 and 50 μg/mL) in the presence of LPS (100 ng/mL) at 37 °C for 24 h. Then, cells were dispensed into 96-well plates, and 100 μL of each supernatant was mixed with the same volume of Griess reagent (1% sulfanilamide, 0.1% naphthylethylenediamine dihydrochloride and 5% phosphoric acid) and incubated at room temperature for 10 min, the absorbance was measured at 540 nm with a Micro-Reader (Molecular Devices). Serum samples were diluted four times with distilled water and deproteinized by adding 1/20 volume of zinc sulfate (300 g/L) to a final concentration of 15 g/L. After centrifugation at 10,000 × *g* for 5 min at room temperature, 100 μL supernatant was applied to a microtiter plate well, followed by 100 μL of Griess reagent. After 10 min of color development at room temperature, the absorbance was measured at 540 nm with a Micro-Reader. By using sodium nitrite to generate a standard curve, the concentration of nitrite was measured form absorbance at 540 nm.

### 3.7. Statistical Analysis

IC_50_ values were estimated using a non-linear regression algorithm (Sigma Plot 8.0; SPSS Inc. Chicago, IL, USA). Statistical evaluation was carried out by one-way analysis of variance (ANOVA followed by Scheffe’s multiple range tests).

## 4. Conclusions

Those five butanolides **1**–**5** exhibited with no significant cytotoxic activity. As to anti-inflammatory activity, compounds **3** and **4** are stronger than the other three butanolides **1**, **2**, and **5**. The active site may result from the conjugation between the γ-lactone and olefinic functionalities, despite the *E-* or *Z*-form, although compound **5** also possessed conjugation of γ-lactone and olefinic functionalities, it showed no significant active. Therefore, the terminal vinyl group is an essential functionality for anti-inflammation activity by the comparison of structure of compounds **1**–**5**.
